# High prevalence of *Pfcrt* 76T and *Pfmdr1* N86 genotypes in malaria infected patients attending health facilities in East Shewa zone, Oromia Regional State, Ethiopia

**DOI:** 10.1186/s12936-022-04304-5

**Published:** 2022-10-07

**Authors:** Jifar Hassen, Gezahegn Solomon Alemayehu, Hunduma Dinka, Lemu Golassa

**Affiliations:** 1 Department of Applied Biology, School of Applied Natural Science, Adama Science and Technology University, P. O. Box 1888, Adama, Ethiopia; 2 Research and Community Service Center, College of Health Science, Defense University, P. O. Box 1419, Bishoftu, Ethiopia; 3 Aklilu Lemma Institute of Pathobiology, Addis Ababa University, P. O. Box 1176, Addis Ababa, Ethiopia

**Keywords:** *Plasmodium falciparum*, Anti-malarial drug resistance, *Pfcrt*, *Pfmdr1*, Ethiopia

## Abstract

**Background:**

*Plasmodium falciparum* resistance to series of anti-malarial drugs is a major challenge in efforts to control and/or eliminate malaria globally. In 1998, following the widespread of chloroquine (CQ) resistant *P. falciparum*, Ethiopia switched from CQ to sulfadoxine–pyrimethamine (SP) and subsequently in 2004 from SP to artemether–lumefantrine (AL) for the treatment of uncomplicated falciparum malaria. Data on the prevalence of CQ resistance markers after more than two decades of its removal is important to map the selection pressure behind the targets codons of interest. The present study was conducted to determine the prevalence of mutations in *Pfcrt* K76T and *Pfmdr1* N86Y codons among malaria-infected patients from Adama, Olenchiti and Metehara sites of East Shewa zone, Oromia Regional State, Ethiopia.

**Methods:**

Finger-prick whole blood samples were collected on 3MM Whatman ® filter papers from a total of 121 microscopically confirmed *P. falciparum* infected patients. Extraction of parasite DNA was done by Chelex-100 method from dried blood spot (DBS). Genomic DNA template was used to amplify *Pfcrt* K76T and *Pfmdr1* N86Y codons by nested PCR. Nested PCR products were subjected to *Artherobacter protophormiae-I* (*APoI*) restriction enzyme digestion to determine mutations at codons 76 and 86 of *Pfcrt* and *Pfmdr1* genes, respectively.

**Results:**

Of 83 *P. falciparum* isolates successfully genotyped for *Pfcrt* K76T, 91.6% carried the mutant genotypes (76T). The prevalence of *Pfcrt* 76T was 95.7%, 92.5% and 84.5% in Adama, Metehara and Olenchiti, respectively. The prevalence of *Pfcrt* 76T mutations in three of the study sites showed no statistical significance difference (χ^2^ = 1.895; P = 0.388). On the other hand, of the 80 *P. falciparum* samples successfully amplified for *Pfmdr1*, all carried the wild-type genotypes (*Pfmdr1* N86).

**Conclusion:**

Although CQ officially has been ceased for the treatment of falciparum malaria for more than two decades in Ethiopia, greater proportions of *P. falciparum* clinical isolates circulating in the study areas carry the mutant 76T genotypes indicating the presence of indirect CQ pressure in the country. However, the return of *Pfmdr1* N86 wild-type allele may be favoured by the use of AL for the treatment of uncomplicated falciparum malaria.

**Supplementary Information:**

The online version contains supplementary material available at 10.1186/s12936-022-04304-5.

## Background

Despite the reduction of malaria-related morbidity and mortality in recent years, there are 241 million estimated cases and nearly 627 thousand deaths in 2020. The World Health Organization (WHO) African Region alone contributed to 95% and 96% of the estimated global malaria cases and deaths, respectively. Children under 5 years of age are the most risk group, accounting for 77% of all malaria deaths globally in 2020. *Plasmodium falciparum* is the major causes of disease and deaths in this region [1].

*Plasmodium falciparum* developed resistance to every class of anti-malarial drugs to date [2]. Chloroquine resistance (CQR) has been associated with mutations in *P. falciparum chloroquine resistance transporter* (*Pfcrt*) gene and in *P. falciparum multidrug resistance 1* (*Pfmdr1*) gene; however the former is a strong predictor of CQR [3, 4]. In vitro and in vivo studies indicated that substitutions of charged lysine (K) amino acid by uncharged amino acid threonine (T) at amino acid position 76 (K76T) is a primary marker of CQR [3, 4]. Other mutations at amino acid position 72, 74, 75, 97, 152, 163, 220, 271, 326, 356 and 371 have been also identified in CQR *P. falciparum* in different geographical regions [4, 5]. CQR strains of *P. falciparum* originating from CVMNK (CQ sensitive haplotype) are also characterized by CVIET (mostly in Africa) and SVMNT (Asia and South America) haplotypes spanning codons 72–76 of the *Pfcrt* gene [6, 7].

Five point mutations in *Pfmdr1* gene at positions 754, 1049, 3598, 3622 and 4234 result in amino acid changes at codons 86 (N86Y), 184 (Y184F), 1034 (S1034C), 1042 (N1042D) and 1246 (D1246Y), respectively [8]. These mutations have been implicated in increase resistance to CQ, amodiaquine, mefloquine (MQ), quinine (QN) and halofantrine [9]. Out of these mutations N86Y appears to be the most important as it alters the transport activity of the protein and has been implicated as high resistance to CQ [10]. Meanwhile this mutation increases susceptibility to a number of anti-malarial drugs including lumefantrine (LUM), MQ and dihydroartemisinin [9]. Strong linkage disequilibrium was observed between *Pfmdr1* N86Y and *Pfcrt* K76T alleles in CQR isolates of *P. falciparum* [8]. Amplification of the *Pfmdr1* gene also leads to decreased drug sensitivity to QN, LUM and artemisinin [11].

In Ethiopia, CQ treatment failure for *P. falciparum* and *P. vivax* was reported in 1996 [12]. Then, due to widespread of CQ resistant *P. falciparum*, the country changed its treatment policy from CQ to sulfadoxine–pyrimethamine (SP) in 1998 which in turn was replaced by artemether–lumefantrine (AL) for the treatment of uncomplicated falciparum malaria in 2004; however CQ is retained and still continued to be used as a first-line treatment for vivax malaria [6, 13]. Molecular studies from Ethiopia have showed high prevalence of *Pfcrt* K76T mutation that ranged from 41.5 to 100% [6, 13, 14]. However, only one study has showed the return of CQ sensitive *P. falciparum* with 84% of *Pfcrt* K76 sensitive alleles [15]. On the other hand, in Ethiopia, AL pressure might result in directional selection of *Pfmdr1* variants N86, 184 F and D1246 due to LUM partner drug pressure [6, 13–15]. Therefore, it is important to continue monitoring the prevalence of these markers (*Pfcrt* and *Pfmdr1*) that confer resistance to CQ in the hope of possible re-emergence of CQ sensitive *P. falciparum* as previously observed in Malawi [16]. Due to co-existence of *P. falciparum* and *P. vivax*; mixed infections of both parasites are common in Ethiopia [17] that favor the presence of indirect but sustained CQ drug pressure. Therefore, the aim of the present study was to investigate the prevalence of genotypic resistance markers for CQ (*Pfcrt* K76T and *Pfmdr1* N86Y) in *P. falciparum* isolates from East Shewa zone, Oromia Regional State, Ethiopia.

## Methods

### Description of study sites

**Fig. 1:**
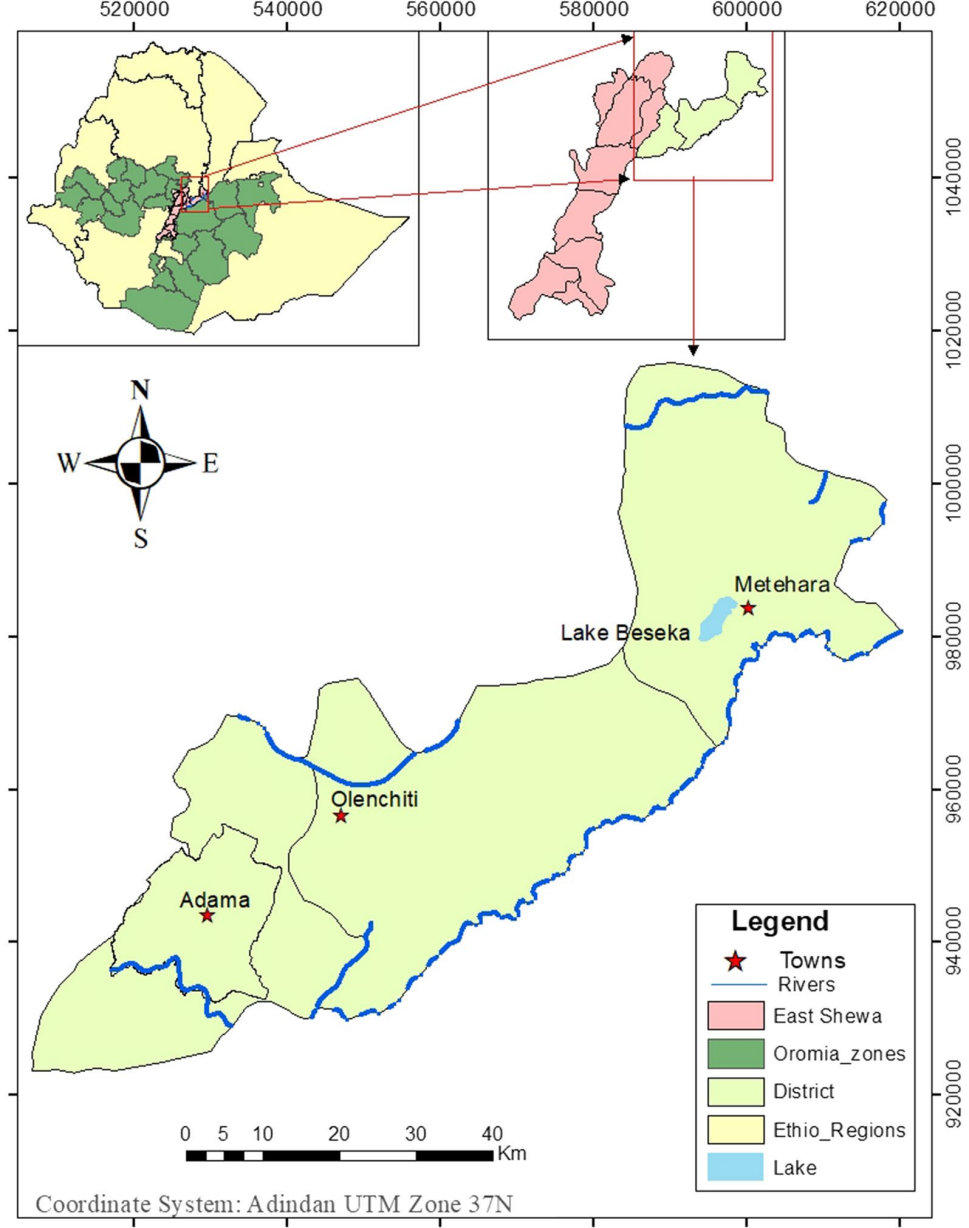
Map of the study area. Arc GIS version 10.8

Cross-sectional health facility based study was conducted at Adama (8° 32′ 31″ N and 39° 16′ 33″ E), Olenchiti (8° 38′ 16″ N and 39° 24′ 19″ E) and Metehara (8° 86′ 95″ N and 39° 92′ 02″ E) health facilities of East Shewa zone, Oromia Regional State, Ethiopia. Adama the capital town of East Shewa zone is found at around 99 km from Addis Ababa, the capital city of Ethiopia. Olenchiti and Metehara towns are found to the East of Adama town at about 25 and 91 km, respectively (Fig. 1). The current study was conducted at purposively selected health facilities due to their physical accessibility to Adama malaria research and diagnostic centre to conduct membrane-feeding assay for another experiment.

The areas are characterized by low altitude (< 2000 m above sea level), heavy rainfall pattern from June to August followed by major transmission of malaria from September to December and short rains of February and March contributing for minor malaria transmission from April to May [18]. Geographical location of the study areas in Great Rift Valley, at low altitude, with rainfall pattern and average annual temperature of 16–32 °C make the areas favorable environments for breeding of *Anopheles* mosquitoes, of which *Anopheles arabiensis* is the major malaria vector [19]. Both *P. falciparum* and *P. vivax* co-exist in the area as it is common in different parts of the country.

### Blood sample collections and processing

Blood samples from one hundred twenty-one microscopically confirmed *P. falciparum* infected patients were collected between October, 2019 and September, 2021. Inclusion criteria include patients who presented with uncomplicated falciparum malaria and history of fever onset since 24 h of clinical examination. Blood samples were collected after obtaining consents from *P. falciparum* infected patients or their parents or legal guardians in case of children less than 18 years of age. After consent was obtained patients sex, age and residency were recorded into log-book. Finger-prick blood samples were collected onto microscope slides and Whatman-3MM ® filter papers by experienced laboratory technologists. Filter papers were allowed to air dry in horizontal position at room temperature for at least 4 h away from dust, insects and direct sun light. Each filter paper was labelled with patient code and date of collection and kept into zip-locked plastic bags with silica gels to protect the specimens from moisture. These zip-locked plastic bags then packed in a larger plastic bags and stored in refrigerator at 2–8 °C for short period in the field and further stored in deep freezer at − 20 °C at Aklilu Lemma Institute of Pathobiology for long period until DNA analysis for molecular study.

### Microscopic diagnosis and parasite counts

Microscopy diagnosis of both Giemsa-stained thick and thin blood films was conducted according to WHO protocol [20]. At each health facilities slides were read by two experienced laboratory technologists to confirm species identification for *P. falciparum*. In addition slides re-examinations and parasite counts were performed by two WHO certified malaria microscopists at Adama malaria research and diagnostic centre. Further, asexual stages of the parasite per micro-litre (µl) was determined by counting the number of parasites against 200 white blood cells (WBCs) on a thick blood film assuming a total of standard WBCs count of 8000/µl of blood. Density of the parasite was determined by using the following formula [21]:

$$\text{Parasite density}/ \upmu \text{l} = \frac{\text{No. of asexual parasites counted} \times 8 \times 10^3 \,\text{WBCs}/ \upmu \text{l}}{200 \text{WBCs}}$$

For comparison with *Pfcrt* K76T wild and mutant alleles, parasitaemia was categorized as low (< 1000 parasites/µl of blood), moderate (1000–9999 parasites/µl of blood) and high (≥ 10,000 parasites/µl of blood) [22].

## Molecular analysis

### DNA extraction

*Plasmodium falciparum* genomic DNA was extracted from dried blood spot (DBS) by Chelex extraction method as described elsewhere [19]. Extracted DNA was transferred into 0.5 ml tubes and stored at − 20 °C until further use.

### Amplification of the *Pfcrt* gene

A fragment from *Pfcrt* gene encompassing 76th codon was successively amplified for 83 DNA samples. Nested polymerase chain reaction (PCR) protocol adapted and modified from the work of Mulenga et al. [23]. Briefly, in primary PCR, 20.0 µl reaction mixture consisting of 11.6 µl nuclease free water, 0.2 µl of each forward and reverse primers, 4.0 µl 5X FIREPol® Master Mix (Solis BioDyne) and 4.0 µl of DNA template was used. PCR was conducted in a thermo cycler (VWR) Schmidt, Germany. Amplification was performed under the following conditions: Hot start denaturations at 94^o^C for 3 min, followed by 30 cycles of 94 °C for 30 s, 56 °C for 30 s and 65 °C for 1 min and final elongation at 65 °C for 3 min. Then, the primary PCR was followed with nested PCR. Twenty microlitre reaction mixture consisting of 14.6 µl nuclease free water, 0.2 µl of each forward and reverse primers (Additional file 1), 4.0 µl 5X FIREPol Master Mix (Solis BioDyne) and 1.0 µl of DNA template was used. The nested PCR was conducted as follows: Hot start denaturation at 94 °C for 5 min, followed by 40 cycles of 94 °C for 30 s, 52 °C for 30 s and 65 °C for 1 min and lastly, final elongation at 65 °C for 3 min.

### Amplification of *Pfmdr1* gene

*Pfmdr1* gene was amplified to identify mutation at codon N86Y according to the protocol described by Fontecha et al. [24] with certain modifications. Briefly, both primary and nested PCRs were conducted in a total reaction volume of 18.0 µl consisting of 11.4 µl ddH_2_O, 1.0 µl of each forward and reverse primers (Additional file 1), 3.6 µl 5X FIREPol Master Mix and 1.0 µl DNA template. In primary PCR, the target gene was amplified starting with hot start denaturation at 95 °C for 3 min, followed by 20 cycles of 95 °C for 30 s, 52 °C for 30 s and 72 °C for 1 min and lastly, final extension at 60 °C for 5 min. Nested PCR amplification was conducted beginning with hot start denaturation at 95 °C for 3 min, followed by 25 cycles of 95 °C for 30 s, 54 °C for 30 s, and 72 °C for 60 s and final extension at 72 °C for 5 min.

For both *Pfcrt* K76T and *Pfmdr1* N86Y codons, PCR conditions were conducted along with nuclease free water (negative control) instead of parasite DNA, 3D7 (CQ sensitive) and K1 (MRA-159G, CQ resistant) clones of *P. falciparum* genomic DNA (positive controls). The two strains were provided by Ethiopian Public Health Institute and Aklilu Lemma Institute of Pathobiology, respectively. Five microlitre of nested PCR products were resolved in 2.0% and 2.5% agarose, type I gel (Sigma Alorich, USA) stained with ethidium bromide (EtBr) respectively, for *Pfcrt* K76T and *Pfmdr1* N86Y codons. Electrophoresis conditions were conducted at 100 volts for 45 min in a Thermo Scientific apparatus filled with 0.5X TBE buffer solution (BIONEER Corp, Korea). Then, DNA bands were observed through ultra-violet (UV) trans-illuminator (USA). The band sizes were estimated by relating with 100 bp DNA ladder marker (Bionexus). The remaining 15 µl and 13 µl nested PCR products of *Pfcrt* K76T and *Pfmdr1* N86Y, respectively were stored at 2–8 °C until used for genotyping by restriction fragment length polymorphism (RFLP).

### Genotyping of *Pfcrt* K76T and *Pfmdr1* N86Y codons by RFLP of PCR products

Following amplification of the two codons, genotyping by PCR-RFLP was made by digesting the fragments with APoI restriction enzyme (New England Biolabs). Nested PCR products were used without purification procedures as this will represent common situations in malaria settings [25]. Restriction digestion was conducted according to the manufacturer’s protocol. Restriction enzyme digestion was done in 20.0 µl of reaction mixture containing 12.5 µl nuclease free water, 5U ApoI restriction enzyme, 2.0 µl NEBuffer r3.1 and 5 µl nested PCR products. Known positive controls (*Pf*3D7, wild-type and K1, mutant type) and uncut DNA samples were included in the reactions. The enzyme cuts *Pfcrt* 76K and *Pfmdr1* N86 but not *Pfcrt* 76T and *Pfmdr1* 86Y alleles. Cleaved PCR products (bands) of *Pfcrt* K76T and *Pfmdr1* N86Y codons were then visualized with UV trans-illuminator after electrophoresis using 2.0% and 2.5% agarose gel, respectively.

### Amplifications of *merozoite surface protein-1 (msp-1)* gene

For the subsets of samples that amplifications of *Pfcrt* and *Pfmdr1* genes were not successful, *msp1* gene amplification was made. Thirty-eight samples which were microscopically detected as *P. falciparum* were not amplified particularly for *Pfcrt* K76T codon. In order to verify the presence of intact genes and quality of DNA for those samples, nested PCR targeting *msp-1* of polymorphic region (block-2) was performed as described elsewhere [19].

### Statistical analysis

Data were first entered into Microsoft Office Excel ® 2007 (Microsoft Corporation). Comparisons of prevalence of 76T allele and parasite densities across study sites and associations of K76 and 76T alleles with parasite density were performed using Pearson’s Chi-square test of Statistical Package for Social Science (SPSS) (IBM Inc., Chicago, IL, USA) version 26. Fisher exact test was used to test correlation between *Pfcrt* 76T alleles and age groups. Statistical significance was set at a P-value < 0.05.

## Results

### Socio-demographic and parasitological characteristics of study population

Eighty- three and eighty samples were successfully analysed for *Pfcrt* K76T and *Pfmdr1* N86Y codons, respectively. The patients were aged between 1 and 68 years and the mean age was 23 ± 14.7 years (interquartile range: 13–30). Seventy-percent of the patients were adults older than 15 years of age. Among patients involved in the study 65% and 35% were males and females, respectively. Parasite density ranged from 79 to 157,538 asexual stages/µl of blood, with a parasitaemia geometric mean of 5366 or median 6579 asexual parasites/µl (Table 1).

**Table 1:** Socio-demographic and parasitological characteristics of the patients recruited in the study (n = 83)

Variables	Values n (%)
Sex	
Males	54 (65)
Female	29 (35)
Age (Years)	
Mean (SD)	23 ± 14.7
Range (IQR)	1–68 (13–30)
0–4	7 (8.4)
5–14	18 (21.7)
15–30	37 (44.6)
> 30	21 (25.3)
Parasitaemia level	
Geometric mean parasite density (SD)	5366 (25532.89)
Range of parasite density (IQR)	79–157538 (1902.5–20700)
< 1000	15 (18.1)
1000–9999	38 (45.8)
> 10,000	30 (36.1)

IQR: interquartile range; SD: standard deviation

**Fig. 2:**
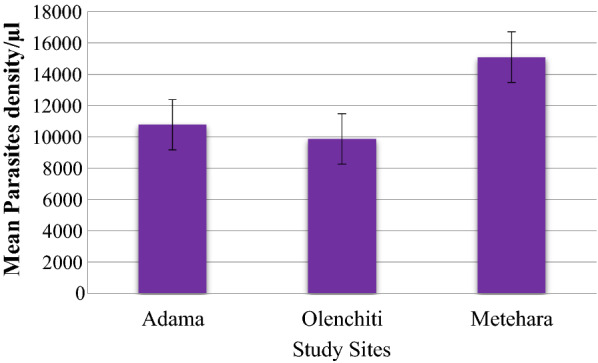
Mean parasites density versus study sites

Average parasites density at Adama, Olenchiti and Metehara sites were 10781.84/µl, 9870.95/µl and 15089.22/µl, respectively; however, the difference is not statistically significant (χ^2^ = 162.75; P = 0.469) (Fig. 2).

### Number of *P. falciparum* clinical samples collected per study site

A total of 121 samples (31 from Adama, 22 from Olenchiti and 68 from Metehara) which were collected from health centres confirmed for *P. falciparum* isolates were allowed for PCR. Eighty three (68.6%) samples were successfully amplified for *Pfcrt* K76T codon; 38 samples were not amplified for the codon. From those samples amplified for *Pfcrt* K76T, 80 were found successfully amplified for *Pfmdr1* N86Y codon.

### Prevalence of *Pfcrt* K76T and *Pfmdr1* N86Ycodons per study site

Digestion of the nested PCR products carrying K76 alleles of *Pfcrt* gene with APoI restriction produced 100 and 45 bps, but remained uncut when 76T mutant allele presents (hence a single band of 145 bp) while digestion of N86 allele of *Pfmdr1* gene with the same restriction enzyme resulted in 239 and 179 bp fragments (denoting wild-type) (Additional file 2).

**Fig. 3:**
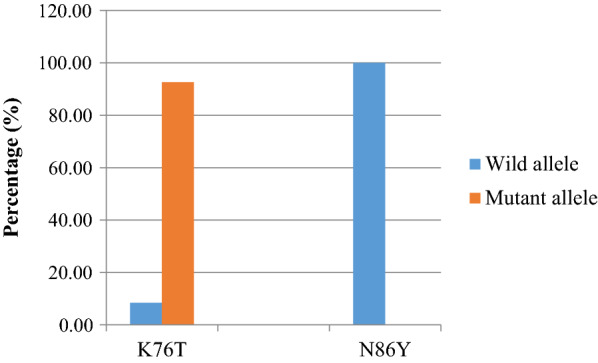
Prevalence of wild and mutant alleles for *Pfcrt* K76T and *Pfmdr1* N86Y codons among *P. falciparum* isolates from Adama, Olenchiti and Metehara study sites

Among 83 *P. falciparum* isolates successfully genotyped for *Pfcrt* K76T, 91.6% and 8.4% mutant- type allele (76T) and wild-type alleles (K76), respectively were detected (Fig. 3). Accordingly, high prevalence of 76T mutant allele was detected at Adama (95.7%), Olenchiti (84.2%) and Metehara (92.7%) study sites with no statistical significant difference (χ^2^ = 1.895; P = 0.388). On the other hand low prevalence of wild-type K76 alleles were detected at each health centres (Table 2). Most of them were detected in *P. falciparum* isolates with moderate parasitaemia level (1000–99,999 parasites/µl). Isolates with 76T allele had a higher mean parasite density of 15,420 parasites/µl while those with K76 allele had a mean parasite density of 7623.14 parasites/µl. However, no significant association was observed between parasite density and allele type (χ^2^ = 76.53; P = 0.620). Eighty nested PCR products of N86Y codons were analysed by restriction enzyme digestion produced N86 (100%) wild-type allele; no 86Y mutant type-allele was detected (Fig. 3).

**Table 2:** Prevalence of *Pfcrt* wild-type and mutant alleles at Adama, Olenchiti and Metehara study areas

Codon	Alleles	Adama (N = 23) n (%)	Olenchiti (N = 19) n (%)	Metehara (N = 41) n (%)	P-value
*Pfcrt* 76	K76	1 (4.3)	3 (15.8)	3 (7.3)	0.388
	76 T	22 (95.7)	16 (84.2)	38 (92.7)	

N is the total number of isolates exposed to restriction digestion by *APoI* enzyme, n represents total number of alleles

### Prevalence of *Pfcrt* 76T allele by gender

Out of 83 isolates successfully genotyped for *Pfcrt* K76T codon, 54 and 29 were males and females, respectively. Males were more represented and contributed for higher prevalence (64.5%) of mutant *Pfcrt* 76T allele. However, the difference was not statistically significant (P = 0.712).

### Prevalence of *Pfcrt* 76T allele across different age groups per study site

**Fig. 4:**
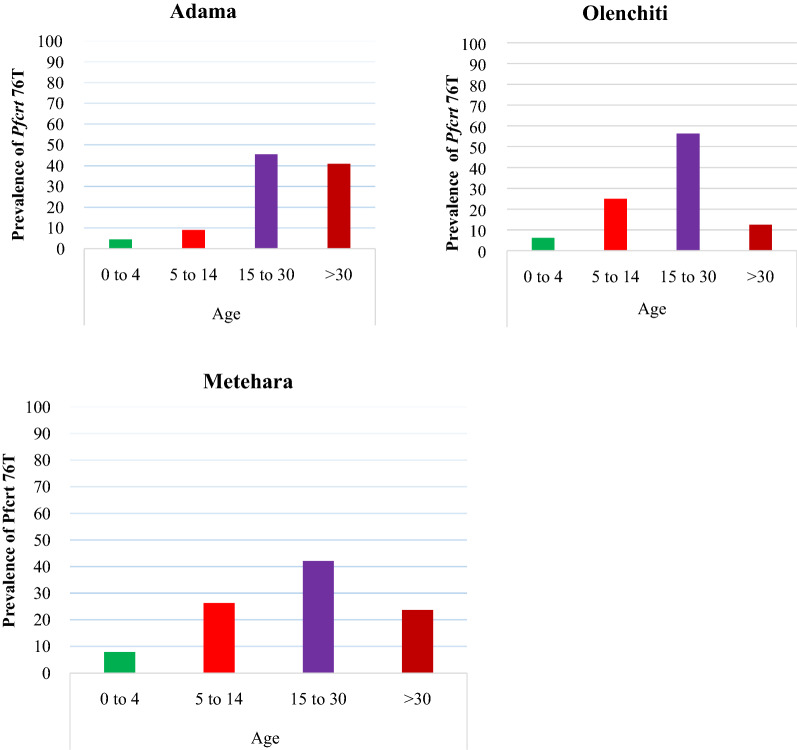
Prevalence of *Pfcrt* 76T CQ-resistant marker among age groups per study sites

Prevalence of *Pfcrt* 76T allele was assessed among age groups at Adama, Olenchiti and Metehara sites. High prevalence of *Pfcrt* 76T alleles was detected among 15–30 age groups with frequencies of 56.3%, 45.5% and 42.1% at Olenchiti, Adama and Metehara sites, respectively (Fig. 4). However, no statistical significance difference of 76T alleles were detected among age groups (Fisher’s exact test, P = 0.69).

### Nested PCR amplification of *Pfmsp1* for samples not amplified for *Pfcrt* K76T and *Pfmdr*1 N86Y

Out of thirty-eight (38) samples which were not amplified especially for *Pfcrt* K76T, 26 (68%) of them were amplified by nested PCR for *Pfmsp1* allelic families of polymorphic region (Block-2). However, still amplification was unsuccessful for 12 of them.

## Discussion

To date, the most important challenge to control and/or eliminate malaria globally is because of emerging and wide-spread anti-malarial drug resistance *Plasmodium* parasites. In this regard a development in drug resistance by *P. falciparum* to series of anti-malarial drugs is the most challenge in fighting malaria for several years [2]. The current study aimed to investigate the prevalence of *Pfcrt* K76T and *Pfmdr1* N86Y codons, in *P. falciparum* isolates collected from Adama, Olenchiti and Metehara sites of East Shewa zone, Oromia Regional State, Ethiopia.

In this study, high frequencies of *Pfcrt* 76T (91.6%) with no significance difference (P = 0.388) was reported from the study areas. Wild-type *Pfcrt* K76 allele was only detected in 8.4% *P. falciparum* isolates. The high proportions of *Pfcrt* 76T allele in this study is in agreement with studies from various parts of Ethiopia [6, 13, 26]. This persistence of high frequency of 76T allele in Ethiopia may be associated with incomplete withdrawal of CQ as it is currently the first-line anti-malarial drug for the treatment of *P. vivax* which accounts nearly for 40% of malaria cases in Ethiopia. Moreover, the persistence of vivax malaria in the form of relapse during dry season may also expose *P. falciparum* to CQ pressure [18]. The dominance of *P. vivax* over *P. falciparum* in the study area was already documented which might contribute for such high frequency of 76T allele [13, 18].

Another potential probable reason for circulations of high frequency of *Pfcrt* 76T mutant allele in the study areas may be because of the existence of mixed infections of *P. falciparum* and *P. vivax* [17]. Misdiagnosis of malaria [27], such as mixed infections, as *P. vivax* and treating patients with CQ was commonly encountered during the study period (unpublished observations) and this may expose *P. falciparum* to indirect CQ pressure selecting for the mutant genotypes.

To the best of our knowledge, in the present study areas there is no base-line data regarding the prevalence of 76T allele at the time when CQ was replaced with AL. However, 2 years later study conducted by Schunk et al. [28] in Southern Ethiopia showed fixation of 76T in parasite isolates in the area. Ten years later, since the implementation of AL, study conducted at Adama by Golassa et al. [6] revealed fixation of 76T mutant allele in parasite population, however; in the current study wild-type K76 allele was recovered in the frequency of 8.4%. In the current low malaria endemic area [29], the mutant allele still may circulate at high frequencies in parasite populations between human host and *Anopheles* vectors in the study areas. In this low malaria transmission setting, lower rate of sexual recombination parasite genotypes may take place in *Anopheles* mosquitoes, which might result in more resistant genotypes to be established in the human host. Therefore, malaria transmission involving multiple genotypes is uncommon in low transmission areas. Conversely, in high malaria transmission areas competition between drug sensitive and drug resistant parasites may slow the spread of drug resistant parasites [23, 30]. Study conducted by Hailemeskel et al. [13] which investigated the prevalence of *Pfcrt* K76T genotype in low and high malaria endemic areas of Ethiopia [29] showed significantly higher prevalence of 76T and K76 alleles in low and high malaria endemic areas, respectively.

In countries like Ethiopia where CQ drug pressure persists for the treatment of vivax malaria, high frequency of the mutant allele is being reported from Yemen [31], Malaysia [22], Nigeria [32], India [33], Bangladesh [34] and Angola [35]. Conversely, progressive decrease in 76T allele and recovery of K76 allele were documented particularly in sub-Saharan African countries such as Malawi [16] Tanzania [36], Kenya [37] and Uganda [38], where AL is being implemented. Though AL is used as first-line treatment for *P. falciparum* in Ethiopia low recovery of parasites harbouring wild-type K76 was detected in the study areas may be because of reasons already described above.

In this study, associations of *Pfcrt* K76T mutation with parasite density, age and gender were investigated. Some studies have associated this mutation with parasite density [22, 31]; while others have found no such associations [39]. In the current study we have found no association of these mutations with parasite density. On the other hand, the detection of high prevalence of this mutation among 15–30 age groups in this study may be explained by more exposure of these groups to mosquito bite and CQ pressure than others. However, no significant association was found between K76T mutation and age of patients which is in agreement with findings of Atrosh et al. [22] and Edogun et al. [40]. Similarly, no positive association of this mutation with gender was detected in this study.

In this study, *P. falciparum* clinical isolates analysed carried the wild-type genotypes for *Pfmdr1* N86. The return to the wild-type genotypes was successful in the gene under report after cessation of CQ and use of AL for the treatment of uncomplicated falciparum malaria in Ethiopia. No single mutant *Pfmdr1* 86Y genotype was detected in the study under report. This finding is comparable with the previous reports from different parts of Ethiopia [6, 13, 15, 41]. Study conducted 2 years later after implementation of AL in Ethiopia showed the dominance of 86Y allele over its wild allele counterpart [28]. The current high prevalence of N86 allele (CQ sensitive) showed the survival advantage of wild-type over the mutant type since the implementation of AL for the treatment of falciparum malaria in Ethiopia as evident the selection of this allele by AL elsewhere [42].

In different African countries after adoption of AL for the treatment of falciparum malaria progressive increase in N86 allele and decline in 86Y allele have been documented [43, 44]. In vivo therapeutic studies have showed that N86 wild-type is more prevalent in recurrent infections following AL treatment [45]. In support of this meta-analysis of thirty-one clinical trials have showed a five-fold risk of parasite recrudescence in patients infected with parasites harbouring wild-type N86 allele following treatment with AL compared to patients infected with 86Y mutant parasites [46]. Similarly, allelic replacement study conducted by Veiga et al. [9] revealed that substitution of N86Y mutation with N86 withstands three-four fold higher LUM (partner drug of artemether) and MQ concentrations compared to 86Y residues. Although there is no clear evidence linking N86 allele with treatment failure, high prevalence of this allele in treatment failures in Angola [47] and parasite tolerance to LUM [9, 42], is a clear warning signal for plausible emergence of resistance against AL.

There was unsuccessful nested-PCR amplification for 38 of the samples; particularly for *Pfcrt* K76T codon as was the case for other studies [48]. However, amplification of *Pfmsp1*gene was successful for 26 of them. This indicated that such parasites may have low concentrated intact DNA that could not be amplified. Still, those 12 samples which were not again amplified for *Pfmsp1* indicated that they may be microscopically false positive for *P. falciparum* [27].

## Limitations of the study

The current study is not without limitations. Some of the limitations of the study are small sample size analysed due to logistic constraints, lack of data of copy number variations of the *Pfmdr1* -86 as parasites possessing multiply copy of the *Pfmdr1* N86 showed decreased susceptibility to MQ, artesunate-amodiaquine and LUM [46] and lack of clinical data on the susceptibility of the parasite to LUM that could be obtained from therapeutic efficacy study.

## Conclusion

In conclusion, the present study gives an update on the prevalence of K76T and N86Y alleles in the study areas. A reversal of K76 wild-type allele was detected in non-significant proportions while a fixation of N86 allele was investigated across the study areas. The data obtained from this study calls for continuous monitoring anti-malarial drug resistance against falciparum malaria to maintain efficacy of AL for the treatment of uncomplicated *P. falciparum* malaria. Based on this assessment we also recommend careful monitoring of CQ usage for *P. vivax* treatment.

## Supplementary Information

**Additional file 1.** Primers sequences used in nested PCR for the amplification of *Pfcrt* and *Pfmdr1* genes.

**Additional file 2.** Representative gel image showing digested nested PCR products. **(A)**: K76T codon cleaved with *APoI* restriction enzyme run in 2.0% agarose gel. L = 100 bp DNA ladder marker, lanes 1–9 are samples, lane 10 is uncut control and lane 11 is wild-type control (*Pf*3D7). **(B)**: N86Y codon cleaved with APoI restriction enzyme run in 2.5% agarose gel. L = 100 bp ladder, Lanes 1–9 are samples, lane 10 is wild-type pos. cont. (3D7), lane 11 is mutant- type pos. cont. (K1), and lane 12 uncut control.

## Data Availability

The datasets used and/or analysed for this study are available from the correspondent author on reasonable request.

## Abbreviations

ACT: Artemisinin-based combination therapy
AL: Artemether–lumefantrine
CQ: Chloroquine
CQR: Chloroquine resistance
DBS: Dried blood spot
LUM: Lumefantrine
MQ: Mefloquine
QN: Quinine
PCR: Polymerase chain reaction
*Pfcrt*: *Plasmodium falciparum chloroquine resistane transporter*
*Pfmdr1*: *Plasmodium falciparum multidrug resistance 1*
RFLP: Restriction fragment length polymorphism
SP: Sulfadoxine–pyrimethamine
SPSS: Statistical package for social science
WBCs: White blood cells
WHO: World Health Organization

## References

[1] WHO (2021). World Malaria Report 2021.

[2] Roux AT, Maharaj L, Oyegoke O, Akoniyon OP, Adeleke MA, Maharaj R (2021). Chloroquine and sulfadoxine–pyrimethamine resistance in sub-Saharan Africa—a review. Front Genet.

[3] Fidock DA, Nomura T, Talley AK, Cooper RA, Dzekunov SM, Ferdig MT (2000). Mutations in the *P. falciparum* digestive vacuole transmembrane protein *Pfcrt* and evidence for their role in chloroquine resistance. Mol Cell.

[4] Djimde A, Doumbo OK, Cortese JF, Kayento K, Doumbo S, Joseph F (2001). A molecular marker for chloroquine resistant falciparum malaria. N Ingl J Med.

[5] Ibrahim ML, Steenkste N, Khim N, Adam HH, Konate L, Coppee JY (2009). Field-based evidence of fast and global increase of *Plasmodium falciparum* drug resistance by DNA-microarrays and PCR/RFLP in Niger. Malar J.

[6] Golassa L, Kamugisha E, Ishengoma DS, Baraka V, Shayo A, Baliraine FN (2015). Identification of large variation in *Pfcrt*, *Pfmdr-1* and *Pfubp-1* markers in *Plasmodium falciparum* isolates from Ethiopia and Tanzania. Malar J..

[7] Ikegbunam MN, Nkonganyi CN, Thomas BN, Esimone CO, Velavan TP, Ojurongbe O (2019). Analysis of *Plasmodium falciparum Pfcrt* and *Pfmdr1* genes in parasite isolates from asymptomatic individuals in Southeast Nigeria 11 years after withdrawal of chloroquine. Malar J.

[8] Shrivastava SK, Gupta RK, Mahanta J, Dubey ML (2014). Correlation of molecular markers, *Pfmdr1*-N86Y and *Pfcrt*-K76T, with *in vitro* chloroquine resistant *Plasmodium falciparum*, isolated in the malaria endemic states of Assam and Arunachal Pradesh, Northeast India. PLoS ONE.

[9] Veiga MI, Dhingra SK, Henrich PP, Straimer J, Gna N, Uhlemann A (2016). Globally prevalent PfMDR1 mutations modulate *Plasmodium falciparum* susceptibility to artemisinin-based combination therapies. Nat Commun.

[10] Singh G, Singh R, Urhekar AD, Rane K (2016). Gene sequence polymorphisms mutations in *Pfmdr-1* and *Pfcrt-o* genes of *Plasmodium falciparum*. Int J Curr Microbiol Appl Sci..

[11] Cui L, Mharakurwa S, Ndiaye D, Rathod PK, Rosenthal PJ (2015). Antimalarial drug resistance: literature review and activities and findings of the ICEMR Network. Am J Trop Med Hyg.

[12] Tulu AN, Webbeg RH, Schellenberg AJS, Bradley DJ (1996). Failure of chloroquine treatment for malaria in the highlands of Ethiopia. Trans R Soc Trop Med Hyg.

[13] Hailemeskel E, Menberu T, Shumie G, Behaksra S, Chali W, Keffale M (2019). Prevalence of *Plasmodium falciparum Pfcrt* and *Pfmdr1* alleles in settings with different levels of *Plasmodium vivax* co-endemicity in Ethiopia. Int J Parasitol Drugs Drug Resist.

[14] Heuchert A, Abduselam N, Zeynudin A, Eshetu T, Loscher T, Wieser A (2015). Molecular markers of anti-malarial drug resistance in Southwest Ethiopia over time: regional surveillance from 2006 to 2013. Malar J.

[15] Mekonnen SK, Aseffa A, Berhe N, Teklehaymanot T, Clouse RM, Gebru T (2014). Return of chloroquine-sensitive *Plasmodium falciparum* parasites and emergence of chloroquine-resistant *Plasmodium vivax* in Ethiopia. Malar J.

[16] Kublin JG, Cortese JF, Njunju EM, Mukadam RAG, Wirima JJ, Kazembe PN (2003). Reemergence of chloroquine-sensitive *Plasmodium falciparum* malaria after cessation of chloroquine use in Malawi. J Infect Dis.

[17] Ketema T, Bacha K, Getahun K, Del Portillo HA, Bassat Q (2021). *Plasmodium vivax* epidemiology in Ethiopia 2000–2020: a systematic review and meta-analysis. PLoS Negl Trop Dis.

[18] Golassa L, White MT (2017). Population – level estimates of the proportion of *Plasmodium vivax* blood-stage infections attributable to relapses among febrile patients attending Adama Malaria Diagnostic Centre. Malar J.

[19] File T, Chekol T, Solomon G, Dinka H, Golassa L (2021). Detection of high frequency of MAD20 allelic variants of *Plasmodium falciparum merozoite surface protein 1* gene from Adama and its surroundings, Oromia, Ethiopia. Malar J..

[20] WHO. Basic Malaria Microscopy. Part I. Learner’s guide, 2nd Edn. Geneva: World Health Organization; 2010.

[21] Nega D, Daniel D, Tefera T, Eshetu T (2015). Prevalence and predictors of asymptomatic malaria parasitemia among pregnant women in the rural surroundings of Arbaminch town. PLoS ONE.

[22] Atrosh WM, Al-Mekhalfi HM, Mahdy MAK, Surin J (2012). The detection of *Pfcrt* and *Pfmdr1* point mutations as molecular markers of chloroquine drug resistance, Pahang, Malaysia. Malar J.

[23] Mulenga MC, Sitali L, Ciubotariu II, Hawela MB, Hamainza B (2021). Decreased prevalence of the *Plasmodium falciparum Pfcrt* K76T and *Pfmdr1* N86Y mutations post-chloroquine treatment withdrawal in Katete District, Eastern Zambia. Malar J.

[24] Fontecha G, Pinto A, Archaga O, Betancourth S, Escober L, Henríquez J (2021). Assessment of *Plasmodium falciparum* anti-malarial drug resistance markers in *Pfcrt* and *Pfmdr1* genes in isolates from Honduras and Nicaragua, 2018–2021. Malar J.

[25] Veiga MI, Ferreira PE, Gil PJ (2006). Multiplex PCR-RFLP methods for *Pfcrt*, *Pfmdr1* and *Pfdhfr* mutations in *Plasmodium falciparum*. Mol Cell Probes..

[26] Mula P, Fernandez-martinez A, Lucio A, De, Ramos JM, Reyes F, Gonzalez V (2011). Detection of high levels of mutations involved in anti-malarial drug resistance in *Plasmodium falciparum* and *Plasmodium vivax* at a rural hospital in Southern Ethiopia. Malar J.

[27] Jaleta F, Garoma G, Gerenfes T (2020). Evaluation of malaria microscopy diagnosis performance in public hospitals of Eastern and Central part of Oromia Region, Ethiopia, 2019. Pathol Lab Med Int.

[28] Schunk M, Kumma WP, Miranda IB, Osman ME, Roewer S, Alano A (2006). High prevalence of drug-resistance mutations in *Plasmodium falciparum* and *Plasmodium vivax* in Southern Ethiopia. Malar J.

[29] MOH. Ethiopia Malaria Elimination Strategic Plan: 2021–2025. Ministry of Health; Addis Ababa, Ethiopia; 2020.

[30] Yobi DM, Kayiba NK, Mvumbi DM, Boreux R, Kabututu PZ, Situakibanza HNT (2020). Molecular surveillance of anti-malarial drug resistance in Democratic Republic of Congo: high variability of chloroquine resistance and lack of amodiaquinoresistance. Malar J.

[31] Al-Mekhlafi AM, Mahdy MAK, Al-Mekhlafi HM, Azazy AA, Fong MY (2011). High frequency of *Plasmodium falciparum chloroquine resistance* marker (*Pfcrt* T76 mutation) in Yemen: an urgent need to re-examine malaria drug policy. Parasit Vectors.

[32] Adam R, Mukhtar MM, Abubakar UF, Damudi HA, Muhammad A, Ibrahim SS (2021). Polymorphism analysis of *Pfmdr1* and *Pfcrt* from *Plasmodium falciparum* isolates in Northwestern Nigeria revealed the major markers associated with antimalarial resistance. Diseases.

[33] Das S, Tripathy S, Chattopadhayay S, Das B, Kar Mahapatra S, Hati AK (2017). Progressive increase in point mutations associates chloroquine resistance: even after withdrawal of chloroquine use in India. IJP Drugs Drug Resist.

[34] Johora FT, Elahi R, Nima MK, Hossain MS, Rashid H, Kibria MG (2021). Persistence of markers of chloroquine resistance in *Plasmodium falciparum* in Bangladesh. Am J Trop Med Hyg.

[35] Ebel ER, Reis F, Petrov DA, Beleza S (2021). Historical trends and new surveillance of *Plasmodium falciparum* drug resistance markers in Angola. Malar J.

[36] Bwire GM, Ngasala B, Mikomangwa WP, Kilonzi M, Kamuhabwa AAR (2020). Detection of mutations associated with artemisinin resistance at K13- propeller gene and a near complete return of chloroquine susceptible falciparum malaria in Southeast of Tanzania. Sci Rep.

[37] Chebore W, Zhou Z, Westercamp N, Otieno K, Shi YP, Sergent SB (2020). Assessment of molecular markers of anti-malarial drug resistance among children participating in a therapeutic efficacy study in Western Kenya. Malar J.

[38] Balikagala B, Sakurai-Yatsushiro M, Tachibana SI, Ikeda M, Yamauchi M, Katuro OT (2020). Recovery and stable persistence of chloroquine sensitivity in *Plasmodium falciparum* parasites after its discontinued use in Northern Uganda. Malar J.

[39] Afoakwah R, Boampong JN, Egyir-Yawson A, Nwaefuna EK, Verner ON, Asare KK (2014). High prevalence of PfCRT K76T mutation in *Plasmodium falciparum* isolates in Ghana. Acta Trop.

[40] Edogun HA, Daramola GO, Ojerinde AO, Esan CO, Adegbuyi AT (2019). Prevalence of mutant alleles responsible for chloroquine resistance among *Plasmodium falciparum* isolates in North Central, Nigeria. J Adv Biol Biotechnol.

[41] Lo E, Hemming-schroeder E, Yewhalaw D, Nguyen J, Kebede E, Zemene E (2017). Transmission dynamics of co-endemic *Plasmodium vivax* and *Plasmodium falciparum* in Ethiopia and prevalence of anti-malarial resistant genotypes. PLoS Negl Trop Dis.

[42] Otienoburu SD, Maiga-Ascofare O, Schramm B, Jullien V, Jones JJ, Zolia YM (2016). Selection of *Plasmodium falciparum Pfcrt* and *Pfmdr1* polymorphisms after treatment with artesunate-amodiaquine fixed dose combination or artemether–lumefantrine in Liberia. Malar J.

[43] Asare KK, Africa J, Mbata J, Opoku YK (2021). The emergence of chloroquine-sensitive *Plasmodium falciparum* is influenced by selected communities in some parts of the Central Region of Ghana. Malar J.

[44] Chidimatembue A, Svigel SS, Mayor A, Aide P, Nhama A, Nhamussua L (2021). Molecular surveillance for polymorphisms associated with artemisinin-based combination therapy resistance in *Plasmodium falciparum* isolates collected in Mozambique, 2018. Malar J.

[45] Malmberg M, Ferreira PE, Tarning J, Ursing J, Ngasala B, Bjorkman A (2013). *Plasmodium falciparum* drug resistance phenotype as assessed by patient antimalarial drug levels and its association with *Pfmdr1* polymorphisms. J Infect Dis.

[46] Venkatesan M, Gadalla NB, Stepniewska K, Dahal P, Nsanzabana C, Price RN (2014). Polymorphisms in *Plasmodium falciparum chloroquine resistance transporter* and *multidrug resistance 1* genes: parasite risk factors that affect treatment outcomes for *Plasmodium falciparum* malaria after artemether–lumefantrine and artesunate–amodiaquine. Am J Trop Med Hyg.

[47] Dimbu PR, Horth R, Candido LM, Ferreira M, Caquece F (2021). Continued low efficacy of artemether–lumefantrine in Angola in 2019. Antimicrob Agents Chemother.

[48] Adamu A, Jada MS, Haruna HMS, Yakubu BO, Ibrahim MA, Balogun EO (2020). *Plasmodium falciparum multidrug resistance* gene-1 polymorphisms in Northern Nigeria: implications for the continued use of artemether–lumefantrine in the region. Malar J.

