# Supplementary material for: High prevalence of Pfcrt 76T and Pfmdr1 N86 genotypes in malaria infected patients attending health facilities in East Shewa zone, Oromia Regional State, Ethiopia
Source: Malar J. 2022 Oct 7;21:286. doi: 10.1186/s12936-022-04304-5 (PMC9547420; doi:10.1186/s12936-022-04304-5)
Supplement: Supplementary file 1 — Additional file 1. Primers sequences used in nested PCR for the amplification of Pfcrt and Pfmdr1 genes. [file 12936_2022_4304_MOESM1_ESM.docx]

Additional file 1. Primers sequences used in nested PCR for the amplification of *Pfcrt* and *Pfmdr1* genes

| Codon | Primer | Sequence 5’-3’ | Amplicon size (bp) | References |
| --- | --- | --- | --- | --- |
| *Pfcrt,* K76T | CRT-1F | CCGTTAATAATA AATACACGCAG | 537 | Djimde et al., (2001) |
|  | CRT-1R | CGGATGTTACAAAACTATAGTTACC |  |  |
|  | CRT-2F | TGTGCT CAT GTG TTT AAA CTT | 145 |  |
|  | CRT-2R | CAAAACTATAGTTACCAATTTTG |  |  |
| *Pfmdr1,* N86Y | MDR1-1F | TTAAATGTTTACCTGCACAACATAGAAAATT | 612 | Fontecha et al., (2021) |
|  | MDR1-1R | CTCCACAATAACTTGCAACAGTTCTTA |  |  |
|  | MDR1-2F | TGTATGTGCTGTATTATCAGGA | 526 |  |
|  | MDR1-2R | CTCTTCTATAATGGACATGGTA |  |  |
