# Supplementary figures and images for: High prevalence of Pfcrt 76T and Pfmdr1 N86 genotypes in malaria infected patients attending health facilities in East Shewa zone, Oromia Regional State, Ethiopia
Source: Malar J. 2022 Oct 7;21:286. doi: 10.1186/s12936-022-04304-5 (PMC9547420; doi:10.1186/s12936-022-04304-5)

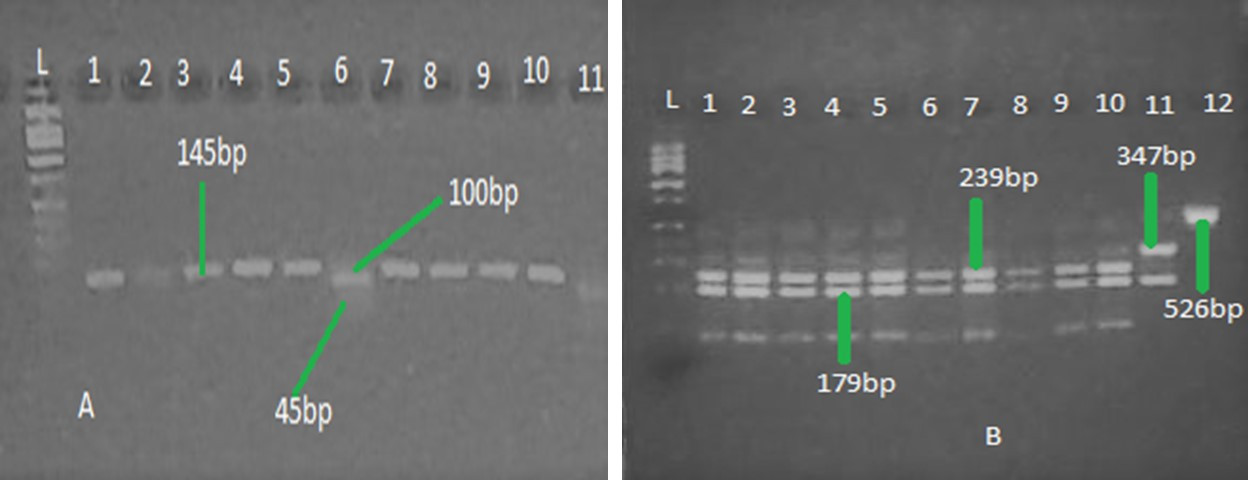

Supplement: Supplementary file 2 — Additional file 2. Representative gel image showing digested nested PCR products. (A): K76T codon cleaved with APoI restriction enzyme run in 2.0% agarose gel. L = 100 bp DNA ladder marker, lanes 1–9 are samples, lane 10 is uncut control and lane 11 is wild-type control (Pf3D7). (B): N86Y codon cleaved with APoI restriction enzyme run in 2.5% agarose gel. L = 100 bp ladder, Lanes 1–9 are samples, lane 10 is wild-type pos. cont. (3D7), lane 11 is mutant- type pos. cont. (K1), and lane 12 uncut control. [file 12936_2022_4304_MOESM2_ESM.jpg]
